# Anatomical Investigation of the Gluteus Medius Muscle Innervation and Its Topographical Correspondence With Myofascial Trigger Points

**DOI:** 10.1155/bmri/7544057

**Published:** 2026-01-18

**Authors:** Roberto Procópio Pinheiro, Daniela Andrea Medina Macaya, Ana Maria Itezerote, Samir Omar Saleh, Flávio Hojaij, Mauro Andrade, Alfredo Luiz Jacomo, Flavia Emi Akamatsu Jacomo

**Affiliations:** ^1^ Department of Surgery, Laboratory of Medical Research, Division of Human Structural Topography, Faculty of Medicine of the University of São Paulo (FMUSP), São Paulo, São Paulo, Brazil, usp.br

**Keywords:** anatomy, gluteus medius muscle, myofascial, nerves, trigger points

## Abstract

**Background and Purpose:**

Hip pain, a common complaint among adults that often causes functional disability, can be caused by femoroacetabular impingement, labral injuries, stress fractures of the femoral neck, avascular necrosis of the femoral head, osteoarthritis of the femoroacetabular joint, hip fractures, greater trochanteric pain syndrome, pathology of the lumbar spine and sacroiliac joint, and myofascial pain syndrome (MPS). MPS is characterized by the presence of hyperirritable nodules, known as myofascial trigger points (MTPs), within muscles and fascia. MTPs limit the range of motion of the joints. Moreover, they induce a local contraction response triggered by mechanical stimulation. The stimulation of MTPs induces pain and sensory changes that can be localized or referred. The MTPs present in the gluteus medius muscle play a role in inducing patellofemoral pain, pain in the lower limbs, anterior region of the knee and thigh, and lower back; however, the anatomy of MTPs remains to be elucidated. This study is aimed at relating the entry points of the superior gluteal nerve into the gluteus medius muscle with the MTPs described in the literature via anatomical dissection.

**Method:**

Twenty gluteus medius muscles of 10 adult cadavers were divided into four areas: posterosuperior, posteroinferior, anterosuperior, and anteroinferior. The distribution of the nerve branches was classified according to these predetermined areas. Statistical analyses were performed using Poisson distribution and logarithmic link function, followed by Bonferroni multiple comparisons (*p* < 0.05).

**Results:**

All areas of the gluteus medius were innervated by the branches of the superior gluteal nerve. A significantly greater number of nerve entry points was observed in Areas II and IV (posterosuperior and anteroinferior, respectively)

**Conclusion:**

The areas of penetration of the superior gluteal nerve correspond to the clinically described MTPs.

## 1. Introduction

Hip pain is a common complaint among adults that often causes functional disability. Chronic hip pain affects 30%–40% of adults who play contact sports [1, 2], whereas it affects 12%–15% of adults aged > 60 years [3, 4]. Picavet and Schouten [5] reported that hip pain affects 10% of the general population and that its prevalence increases with age. Several conditions such as femoroacetabular impingement, labral injuries, stress fractures of the femoral neck, avascular necrosis of the femoral head, osteoarthritis of the femoroacetabular joint, hip fractures, greater trochanteric pain syndrome, pathology of the lumbar spine and the sacroiliac joint [6–8], and myofascial pain syndrome (MPS) (which frequently affect the posterior, lateral, and anterior regions of the hip) have been identified as causes of hip pain [9, 10]. Thus, hip pain may not be related to the joint itself, rather, it may be related to the “hip region,” comprising the groin, buttocks, upper lateral thigh, greater trochanteric region, and iliac crest [11].

Chronic musculoskeletal pain is the primary cause of disability worldwide [12], with MPS accounting for the majority of cases of musculoskeletal pain [13–18]. Few studies have explored the prevalence of MPS in the general population; however, some studies have estimated that MPS is the cause of musculoskeletal pain in 30%–85% of cases [19–23].

MPS, a regional pain disorder that affects individuals of all ages, is characterized by the presence of hyperirritable nodules, known as myofascial trigger points (MTPs), within muscles and fascia [24–27]. MTPs are clinically identified via the palpation of a taut band of muscle or fascia [13, 20, 28]. MTPs limit the range of motion of the related joints. Furthermore, they induce a local contraction response triggered by the mechanical stimulation of certain muscular and fascial areas [13, 28]. This increase in local irritability results in pain and sensory changes that can be localized or referred [25, 29].

Excessive release of acetylcholine in motor endplates has been proposed as the primary factor involved in the development of MTPs [12, 27, 30]. Notably, increased concentrations of acetylcholine in the synaptic cleft, changes in the acetylcholine receptor, and changes in acetylcholinesterase activity are known mechanisms of endplate dysfunction. These mechanisms may explain the increased endplate electrical activity seen in active MTPs [31].

The points of penetration of nerve branches in the muscle belly correspond to the regions where MTPs were clinically described by Travell and Simons [28] and Simons et al. [13, 32–37]. A correlation between the distribution of nerve branching in the trapezius muscle and MTPs was observed in a study, with MTPs and endplates demonstrating a close anatomical relationship [38]. Similarly, a correlation between the location of the MTPs and nerve branching was emphasized in other reports [39, 40]. Ziembicki [41] suggested that nerve entry points are the anatomical basis of the trigger point phenomenon after reviewing the literature on the distribution of innervation and its relationship with MTPs. Furthermore, Ziembicki also suggested that MTPs may form as a result of sensitization of the associated nerve entry points. The International Association for the Study of Pain states that the pathophysiology of MTPs is unclear. However, some morphological changes, neurotransmitters, neurosensory characteristics, electrophysiological characteristics, and motor deficiencies have been implicated in its pathogenesis [42]. These changes may be attributed to defective nerve signaling.

Mehdikhani et al. conducted an electromyographic study in 2022 and demonstrated that MTPs played a role in inducing muscle dysfunction [43]. Ziembicki [41] established patterns of spatial distribution of MTPs and their possible correlation with nerve branching in several different muscles based on the findings of previous studies. The research yielded strong evidence supporting the anatomical correlation between the nerve entry points and MTPs. Previous studies on the trapezius, gluteus maximus, abductor hallucis, masseter, temporalis, and deltoid muscles conducted by our group were also evaluated [32–37], and the methodology employed was deemed reliable.

MTPs have been detected in the muscles of the hip and pelvic region [44–48]. Roach et al. [46] reported that MTPs induce pain, weakness, spasms, and fatigue in the gluteus medius muscle, the main stabilizer of the hip. Moreover, MTPs have been frequently detected in the gluteus medius muscle of patients presenting with patellofemoral pain [49], pain in the lower limbs [50], pain in the anterior region of the knee and thigh [51–53], and lower back pain [28, 45, 47, 50, 54–56].

The gluteus medius, a broad muscle that lies between the gluteus maximus and minimus muscles, covers the outer surface of the ilium between the anterior and posterior gluteal lines. The gluteus medius is located superior to the gluteus minimus and its posterior fibers converge to form a flat tendon inclined inferiorly and anteriorly towards the superoposterior facet of the greater trochanter of the femur. The anterolateral portion of this tendon is directed posteriorly to reach the lateral facet of the greater trochanter [57, 58]. The gluteus medius is the primary hip abductor muscle, and its anterior fibers also assist with the internal rotation of the thigh. The posterior fibers contribute to lateral rotation [46, 59, 60].

The gluteus medius muscle is innervated by the superior gluteal nerve (SGN), which originates from the dorsal branches of the L4, L5, and S1 nerve roots in the sacral plexus. SGN exits the pelvis through the greater sciatic foramen superior to the piriformis muscle and divides into the inferior and superior branches. The superior branch of SGN, which accompanies the superior branch of the deep division of the superior gluteal artery, innervates the gluteus minimus and medius. The inferior branch, which accompanies the inferior branch of the deep division of the superior gluteal artery, innervates the gluteus minimus and medius and ends in the tensor fasciae latae muscle [57, 61].

**Figure 1:**
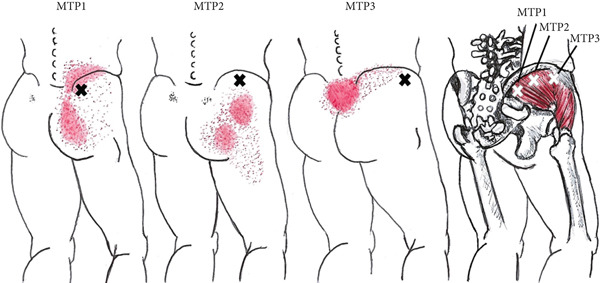
Schematic diagram of pain patterns referring to trigger points (MTPs) (Xs) in the right gluteus medius muscle, based on the location described by Travell and Simons [28]. The essential pain pattern is indicated in solid red. The spread pattern appears dotted.

Travell and Simons [28] reported that MTPs are located at three common sites in the gluteus medius muscle. The MTP1 region corresponds to the region located close to the iliac crest in the posterior portion of the muscle near the sacroiliac joint. MTP1 induces pain and sensitivity in this area, which may extend over a large area of the buttock. The MTP2 region corresponds to the area located immediately below the middle of the iliac crest. The referred pain from MTP2 radiates laterally and may extend posteriorly and laterally to the upper thigh. The MTP3 region corresponds to the area located below the iliac crest close to the anterior superior iliac spine. The referred pain from MTP3 radiates along the iliac crest over the lower lumbar and sacral regions (Figure 1).

The complex pathophysiology of MTPs remains unclear [13, 14, 42]. Therefore, this study is aimed at correlating the branching of the SGN within the gluteus medius muscle with the MTPs already described in the literature through anatomical dissection, thereby providing an anatomical basis for the pathophysiology of MPS. The findings of this study may offer useful information for clinical management and interventional procedures.

## 2. Material and Methods

### 2.1. Ethical Aspects

This study was approved by the Research Ethics Committee of the Faculty of Medicine, University of São Paulo (Research Protocol Number: 105/14).

### 2.2. Anatomical Technique

This study uses the method of Akamatsu et al. [33] and the methods description partly reproduces their wording.

Twenty gluteus medius muscles of 10 adult human cadavers (5 women and 5 men) donated to the Human Structural Topography Discipline of the Department of Surgery of the Faculty of Medicine were dissected. The cadavers were fixed with a 4% phenolic acid and 0.5% formaldehyde solution. Only specimens with no signs of previous surgical manipulation or other visible abnormalities in the regions of interest were included in this study. The dissection was performed as follows: until the branches of the SGN and their entry points into the gluteus medius muscle were exposed. The specimens were placed in the prone position, and the skin was incised from the lower lumbar region to the gluteal fold and lateral gluteal region. The skin was reflected with the subcutaneous cellular tissue and gluteal fascia to expose the muscle. The gluteus maximus muscle was reflected posteriorly to expose the gluteus medius muscle. The gluteus medius muscle was cut from its origin in the iliac bone and reflected to dissect its deep surface to preserve and observe the branching of the SGN following its emergence from the pelvis through the greater sciatic foramen present superior to the piriformis muscle.

### 2.3. Gluteus Medius Muscle Measurements and Quadrant Delimitation

**Figure 2:**
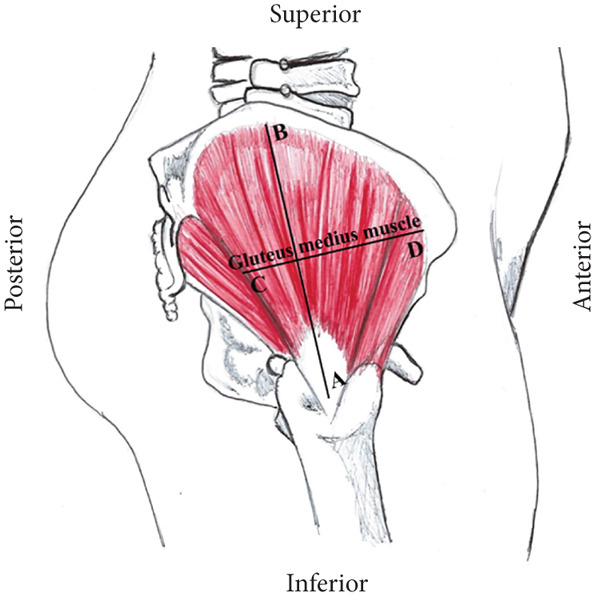
Schematic diagram of the right lateral view of the gluteal region. The dimensions of the gluteus medius muscle according to sex in relation to the AB segments: longitudinal line of the muscle and CD: mid‐transverse line of the muscle.

Morphometric measurements of the following muscular dimensions were acquired: the longitudinal length (AB), defined as the largest muscular dimension extending from the insertion of the tendon of the gluteus medius muscle in the greater trochanter of the femur to the highest point of the origin of the gluteus medius muscle at the iliac crest and the middle transverse length (CD), measured at the midpoint of the longitudinal line involving all transverse extensions of the muscle belly (Figure 2).

**Figure 3:**
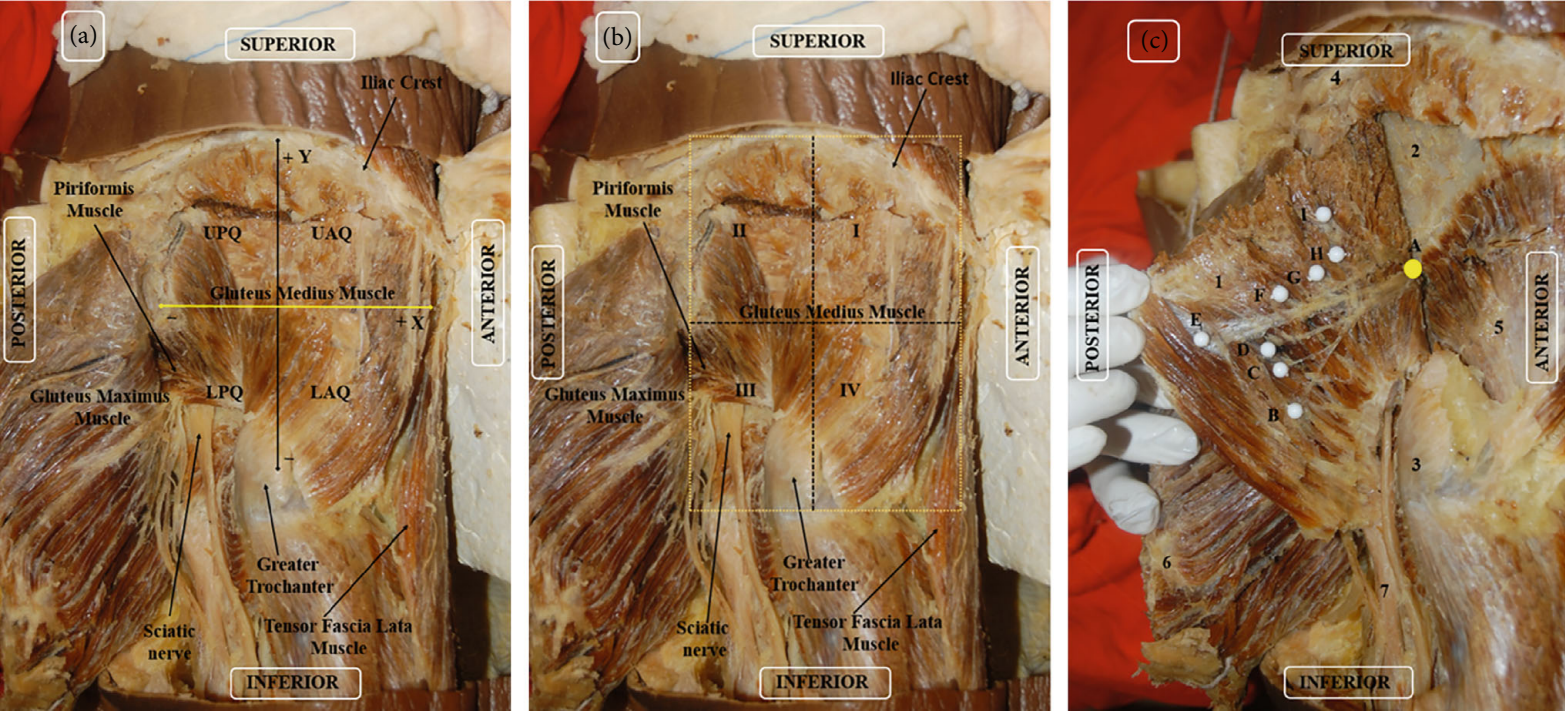
(a) Right lateral view of the gluteal region of the cadaver. The orientation of the abscissa (*x*) and ordinate (*y*) of the right gluteus medius. By convention: upper anterior quadrant (UAQ) with positive ordinate and abscissa; lower anterior quadrant (LAQ) with negative ordinate and positive abscissa; upper posterior quadrant (UPQ) with positive ordinate and negative abscissa; lower posterior quadrant (LPQ) with negative ordinate and abscissa. (b) Right lateral view of the gluteal region of the cadaver. Quadrant of the right gluteus medius muscle to locate the points of penetration of the superior gluteal nerve. CD: middle transverse line separates the upper areas from the lower areas, which are divided into two segments of equal size. The areas are numbered from I to IV. I Areas I and II correspond to the anterosuperior and posterosuperior areas, respectively. Areas III and IV correspond to the posteroinferior and anteroinferior areas, respectively. (c) Cadaver in prone position, right lateral view. 1: gluteus medius muscle repressed; 2: iliac bone; 3: greater trochanter of the femur; 4: iliac crest; 5: gluteus minimus muscle; 6: gluteus maximus muscle; 7: sciatic nerve. A: superior gluteal nerve indicated in yellow; B, C, D, E, F, G, H, and I indicating the branches of the superior gluteal nerve entering the gluteus medius muscle (in white).

The entry points of SGN into the muscle in relation to the middle transverse and longitudinal measurements were determined based on AB and CD, and the Cartesian plane was delimited in the abscissa and ordinate, respectively. The relative values of the penetration point in relation to the mean transverse and longitudinal dimensions were calculated as these values tend to vary depending on the size of the muscle. Thus, these dimensions constitute 100% of the muscle size, with the muscle insertion values making a small contribution. The intersection of the axes was considered as the origin and zero point: superior–anterior quadrant with positive abscissa and ordinate, inferior–anterior quadrant with positive abscissa and negative ordinate, superior–posterior quadrant with negative abscissa and positive ordinate, and inferior–posterior quadrant with abscissa and negative ordinate (Figure 3a).

Four distribution areas were formed to group the data into categories and facilitate clinical correlation, with the middle transverse line separating the upper and lower areas. The areas were numbered from I to IV. Areas I and II correspond to the anterosuperior and posterosuperior areas, respectively. Areas III and IV correspond to the posteroinferior and anteroinferior areas, respectively (Figure 3b).

The dissection was performed from the apparent origin of SGN at the level of the piriformis muscle until it branched and penetrated the gluteus medius muscle. The entry points of the branches of SGN into the muscle were marked using colored pins. Photographic records of all specimens were acquired (Nikon D52). The location of the entry points in relation to the mean longitudinal and transverse axes was measured via a simple division of the values and classified into the areas numbered from I to IV (Figure 3c).

### 2.4. Statistical Analysis

The sample calculation was performed based on the results of the first six cadavers evaluated in a pilot study. The difference between Quadrants II and III (QII and QIII, respectively) was on average 2.17 nerve entry points in the pilot study, with a variability of 1.9 points (SD = 1.9 points). Thus, the sample size was calculated as 20 muscles, assuming a power of 78% and an alpha value of 5%.

Summary measurements (mean, standard deviation, median, and minimum and maximum) were used to describe the quantitative characteristics of the cadavers, whereas absolute and relative frequencies were used to describe the qualitative characteristics of the cadavers.

The muscle characteristics of the cadavers were described according to sex using summary measurements and compared using the Student′s *t*‐test [62]. The muscle measurements acquired for each side were compared using a paired Student′s *t*‐test [62].

The number of nerve entry points in each muscle was described and compared between the sides and quadrants using generalized estimation equations with an exchangeable correlation matrix with a normal and Poisson marginal distribution for the entry points of the muscle nerves and identity link function [63], respectively. Bonferroni multiple comparisons [64] were performed to identify the differences among the quadrants (I–IV). The results are illustrated in tables representing means and standard errors.

All statistical analyses were performed using IBM‐SPSS software for Windows Version 26.0. (IBM Corp.; released 2019. IBM SPSS Statistics for Windows, Version 26.0; Armonk, NY; IBM Corp.). The significance level was set as 5% (*p* < 0.05).

## 3. Results

The ages of the cadavers ranged 32–92 years (mean = 63.3 years). The approximate heights ranged 1.7–1.8 m (mean = 1.75). The weight and body mass index (BMI) ranged 42–85 kg (mean = 69) and 14.5–29.4 kg/m^2^ (mean = 22.5), respectively. Seven of the 10 cadavers were of Caucasian descent; the remaining three were non‐white (Table 1)

**Table 1:** Cadaver characteristics and the results of statistical analyses.

**Variables**	**Cadaver**
	**(** **n** = 10**)**
Age	
Mean ± SD	63.3 ± 20.3
Median (min.; max.)	60 (32; 92)
Sex; *n* (%)	
Female	5 (50)
Male	5 (50)
Race; *n* (%)	
White	7 (70)
Brown	3 (30)
Weight (kg) ^∗^	
Mean ± SD	69 ± 13.5
Median (min.; max.)	69 (42; 85)
Height (m) ^∗^	
Mean ± SD	1.75 ± 0.05
Median (min.; max.)	1.75 (1.7; 1.8)
BMI (kg/m^2^) ^∗^	
Mean ± SD	22.5 ± 4.4
Median (min.; max.)	21.9 (14.5; 29.4)

Abbreviations: BMI, body mass index. SD, standard deviation.

^∗^Information was available for only eight cadavers.

Measurements of the longitudinal (AB) and transverse (CD) muscle dimensions according to sex revealed that the mean right CD in the female cadavers was larger than that in the male cadavers (*p* = 0.016). No significant differences were observed between the other characteristics in terms of sexes or sides (*p* > 0.05) (Table 2).

**Table 2:** Description of gluteus medius muscle measurements according to sex and results of comparisons between sexes and sides of the cadavers.

**Variables**	**Sex**		**Total (** **N** = 10**)**	**p**
	**Female (** **N** = 5**)**	**Male (** **N** = 5**)**		
AB (cm) Right				0.201
Mean ± SD	14 ± 1.8	15.4 ± 1.4	14.7 ± 1.7	
Median (min.; max.)	14.5 (11.5; 16)	15.5 (14; 17)	14.8 (11.5; 17)	
AB (cm) Left				0.948
Mean ± SD	15 ± 1	15 ± 0.9	15 ± 0.9	
Median (min.; max.)	15 (14; 16)	15 (14; 16.5)	15 (14; 16.5)	
*p* ^∗^			0.543	
CD (cm) Left				0.736
Mean ± SD	11.5 ± 1.7	11.2 ± 0.8	11.4 ± 1.3	
Median (min.; max.)	12 (8.5; 13)	11 (10.5; 12.5)	11.8 (8.5; 13)	
CD (cm) Right				0.016
Mean ± SD	13.3 ± 1.7	10.4 ± 1.4	11.9 ± 2.1	
Median (min.; max.)	13.5 (11.5; 15.7)	10.5 (9; 12)	11.8 (9; 15.7)	
*p* ^∗^			0.516	

*Note:* Dimensions of the gluteus medius muscle in relation to segments: AB, longitudinal line of the gluteus medius muscle; CD, middle transverse line.

Abbreviation: SD, standard deviation.

^∗^Paired Student′s t‐test.

The points of entry of SGN into the muscle belly were observed in all quadrants of all cadavers, regardless of sex or side. However, the distribution of the SGN entry points among the quadrants varied significantly (*p* < 0.001) (Table 3).

**Table 3:** Description of the number of nerve entry points in the gluteus medius muscle according to quadrants in the cadavers and results of the comparative test.

**Quadrants**	**Points of entry**		**p**
	**M** **e** **a** **n** ± **S** **D**	**Median (min.; max.)**	
I	1.95 ± 1.47	1.5 (0; 5)	<0.001
II	3.1 ± 1.83	3 (0; 7)	
III	1.1 ± 1.45	0.5 (0; 5)	
IV	3.1 ± 1.68	3 (0; 6)	

*Note:* GEE with Poisson distribution and identity link function, assuming an exchangeable correlation between the quadrants and sides of the muscles.

Abbreviation: SD, standard deviation.

**Figure 4:**
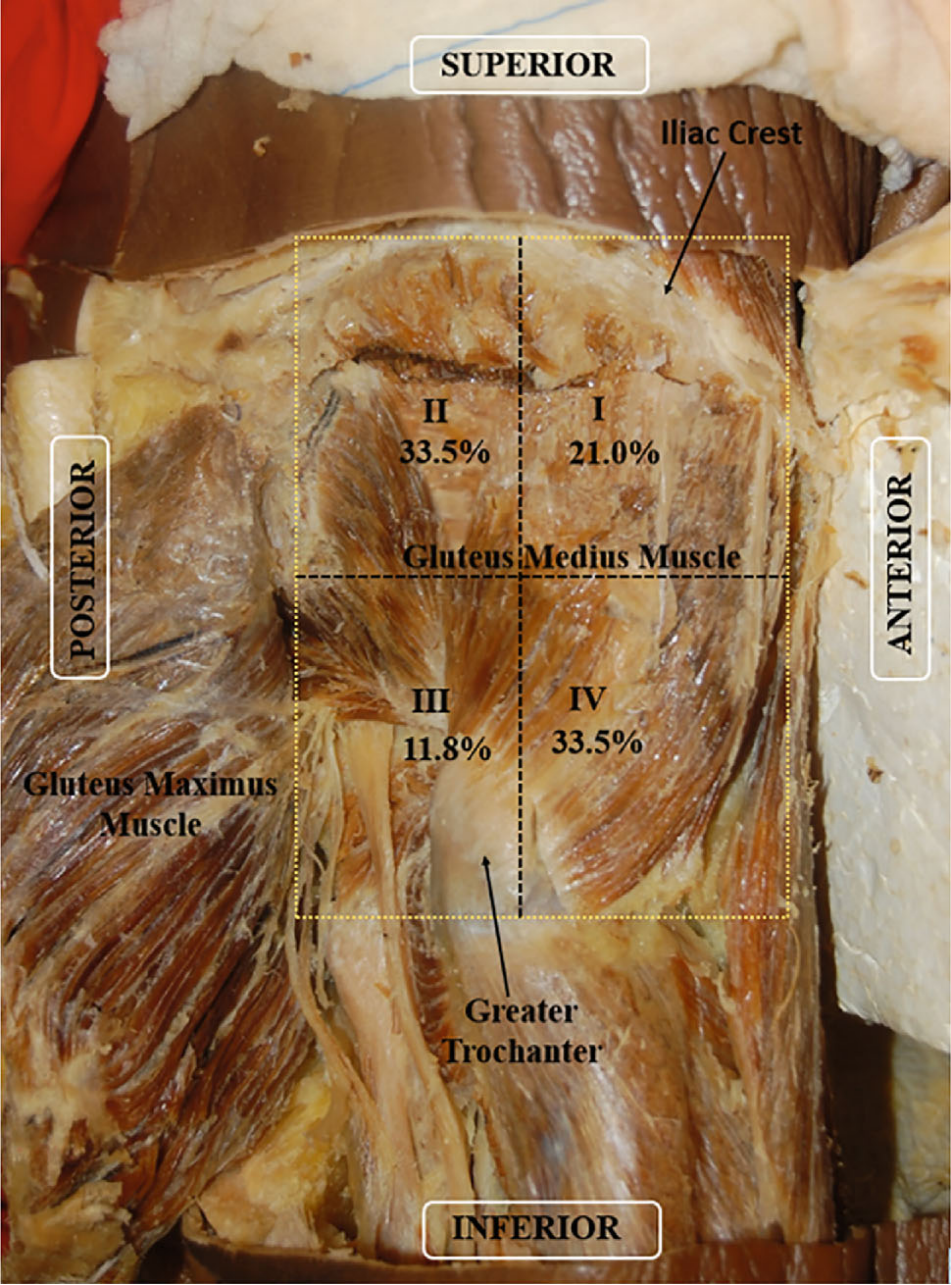
Right lateral view of the gluteal region of the cadaver. Quadrants of the gluteus medius muscle. Percentage of nerve entry points per area. The areas are numbered from I to IV. Areas I and II correspond to the anterosuperior and posterosuperior areas, respectively. Areas III and IV correspond to the posteroinferior and anteroinferior areas, respectively.

The mean number of nerve entry points in the muscle was significantly lower in Quadrant III than those in Quadrants II and IV (*p* < 0.001). Area I had the third highest number of nerve entry points (Table 4 and Figure 4).

**Table 4:** Comparisons between the quadrants of the number of nerve entry points into the gluteus medius muscle.

**Comparison**	**Mean difference**	**Standard error**	**GL**	**p**	**IC (95%)**	
					**Inferior**	**Superior**
II–I	1.15	0.53	1	0.175	−0.24	2.54
II–III	2.00	0.48	1	< 0.001	0.74	3.26
II–IV	0.00	0.59	1	> 0.999	−1.55	1.55
I–III	0.85	0.41	1	0.227	−0.23	1.93
I–IV	−1.15	0.53	1	0.175	−2.54	0.24
III–IV	−2.00	0.48	1	< 0.001	−3.26	−0.74

*Note:* Bonferroni multiple comparisons.

Abbreviations: GL, degree of freedom; IC, confidence interval.

## 4. Discussion

The SGN entry points in the gluteus medius muscle were considered to be closely related to the MTPs inducing painful disorders of the buttock region in this study. The same reasoning was followed in the previous studies conducted by our group on the innervations of the trapezius, gluteus maximus, abductor hallucis, masseter, temporal, and deltoid muscles [32–37]. Previous studies have suggested some variations in terms of the distribution areas; however, the description of MTPs reported by Travell and Simons [28] was considered in the present study as there were no precise previous data regarding their locations in the gluteus medius muscle.

SGN entry points were observed in all quadrants of the muscles. Region IV had the greatest number of entry points, with their frequency decreasing sequentially in the following order: Region II > Region I > Region III.

The MTPs described by Travell and Simons for the gluteus medius (1983) correspond to Regions I and II (anterosuperior and posterosuperior areas, respectively), as observed in this study. Rozenfeld et al. [53] evaluated the association between anterior knee pain and the prevalence of MTPs in the hip and thigh muscles and reported that MTPs were present in the distal and anterior part of the gluteus medius muscle, close to the greater trochanter of the femur. This finding does not follow the pattern of pain described by Travell and Simons [28]; however, Travell and Simons [28] have reported occasional occurrences of MTPs in other parts of the gluteus medius muscle. The MTPs reported by Rozenfeld et al. [53] correspond to Area IV (anteroinferior area) in this study, which presented the same number of nerve penetration points as Area II.

Tomlinson et al. [65] conducted a systematic review and meta‐analysis and provided a comprehensive synthesis of hip joint capsule innervation from anatomical studies [65]. The hip capsule is innervated by SGN and the other nerves of the lumbosacral plexus. The cadaveric study conducted by Nagpal et al. [66] revealed that small articular branches of SGN innervate the hip. A descending course of the nerve from its posterior origin towards the hip joint capsule, located in a region closely related to the lower part of the gluteus medius muscle, was reported in both of these studies. This area corresponded to Area IV in this study.

Duparc et al. [67] and Pérez et al. [68] reported that the great trochanter of the femur is a reliable surface marker of the course of SGN. Another cadaveric study conducted by Akita et al. [69] revealed that the anterior fibers of the gluteus medius muscle are innervated by the branches of SGN. This finding suggests that the medial rotation of the hip and the action of the anterior fibers of the gluteus medius muscle influence the course and distribution of the SGN branches. A greater number of nerve penetration points was observed in this portion of the muscle in this study, which corresponds to Area IV.

The gluteus medius is inserted inferiorly into the greater trochanter. Patients frequently report experiencing pain on the lateral and posterior surfaces of the greater trochanter, which is also associated with tenderness in response to deep pressure. Ultrasonography and magnetic resonance imaging can be used to diagnose enthesopathy related to the gluteal tendons or their insertion [70]. Travell and Simons [28] reported that inflammation of the anterior trochanteric bursa of the gluteus medius can induce pain and sensitivity in the same region of the greater trochanter. However, this type of pain must be distinguished from pain induced by MTPs in the gluteus medius muscle in response to the excitation of the nerve and taut muscle bands. Differential diagnoses can be made based on the findings of direct examination of the clinically described MTPs in this area.

Several previous studies have hypothesized that MTPs originate in the peripheral nerves [13, 28–31, 71–75]. This hypothesis was first suggested by the French doctor François Louis Isidore Valleix in his study published in 1841, which described areas of the body wherein these points can be found. In addition to narrow nerve passageways such as the intervertebral foramina to the skin, MTPs were also observed in areas with compression of a superficial nerve by bones, sites of nerve branching, and nerve endings in this study [76]. However, this study did not consider nerve entry points into the muscle belly among the possibilities of pain generation.

Some of the first reports correlating the spatial location of nerve entry points into the muscle and MTPs were published by our group [32]. The locations at which the nerve penetrated the trapezius muscle were coincident with the MTPs described by Travell and Simons in our previous studies. Subsequently, similar studies on the gluteus maximus, abductor hallucis, masseter, temporal, and deltoid muscles were published by our group. These anatomical studies revealed similar results, indicating a close relationship between the areas of nerve penetration and the MTPs described in the literature [34–37].

Previous studies on the distribution of intramuscular nerves have suggested that dysfunction of the innervation zone may be responsible for the development of trigger points [38, 40]. The anatomical substrate may help localize MTPs, thereby guiding therapeutic intervention.

The topographic relationship between nerve branching and MTPs strongly suggests that the nerve entry points and MTPs coincide. Identification of the SGN branching pattern may aid in the clinical identification of MTPs and target spots for therapeutic approaches for painful pelvic girdle disorders involving the gluteus medius muscle. The correspondence between the location of the MTPs clinically described in MPS and the anatomical topography of the SGN entry points in the gluteus medius muscle may be a notable explanation for the triggered activity of MTPs. These findings and the clinical description of MTPs provided by Travell and Simons [28] reinforce the theory that MTPs are caused by excessive release of acetylcholine in the endplates.

A consistent anatomical relationship was observed between the entry points of the SGN branches into the gluteus medius and its MTPs in this study. Division of the gluteus medius muscle into four areas to locate the SGN penetration points revealed that the quadrants were innervated differently, considering the number of nerve entry points in each area. A similar methodology was used by Akamatsu et al. [33] to evaluate the location of the points of penetration of nerve branches in the gluteus maximus muscle via anatomical dissection.

Considering the anatomy of the gluteus medius muscle, the anatomical findings of this study are similar to the location of the MTPs described by Travell and Simons [28], except for Area IV, wherein the presence of MTPs could not be confirmed. However, Rozenfeld et al. reported the presence of MTPs in the distal and anterior parts of the gluteus medius muscle [53]. This information corroborates the findings of this study. Our previous anatomical studies of the trapezius, gluteus maximus, abductor hallucis [34], masseter [35], temporal [36] and deltoid muscles [37] revealed results similar to those of the present study, that is, a relationship with the MTP regions clinically described by Travell and Simons [28]. Thus, considering that the findings of this study are consistent with those of the study by Akamatsu, the MTPs are related to the nerve entry points, despite being present in different muscles.

This study revealed that Areas II (posterior and superior) and IV (anterior and inferior) were the most innervated. Wu et al. reported that SGN enters the gluteus medius muscle and travels in the superior, superolateral, and inferolateral directions, forming a dense zone of arc‐shaped nerves [77]. This dense zone corresponds to Areas II and IV in the present study.

Travell and Simons [28] reported that MTPs in the superior and posterior regions of the gluteus medius muscle produce pain and sensitivity close to the iliac crest, in the posterior portion of the muscle, and close to the sacroiliac joint. This pain can also extend over a large part of the buttocks, radiate more laterally towards the gluteus medius region, and extend posteriorly and laterally to the upper part of the thigh. These findings correspond to Area II. The MTPs in the upper and anterior parts of the muscle radiate pain along the iliac crest, primarily over the lower lumbar region and bilaterally over the sacrum, corresponding to Area I in the present study. This area was less innervated than Areas II and IV. The number of points observed in Area IV, similar to the number of branches directed to Area II, may be related to the course of SGN [65, 67–69, 77–79]. Area III had the smallest number of nerve entry points in the gluteus medius.

Wu et al. reported a greater concentration of nerve branches in the superior, superolateral, and inferolateral areas of the gluteus medius, suggesting that these sites are most suitable for entry into the gluteal muscle [77]. Although a statistical analysis of nerve distribution was not performed in their study, their results are similar to those observed in this study.

Based on current knowledge, the greater concentration of nerve entry points in Areas II and IV may be attributed to the route of the nerve. Notably, the greater number of nerve penetration points in Areas II and IV justifies the clinical relevance of these regions in the pathophysiology of myofascial pain in the gluteus medius muscle.

The transverse dimension (CD) of the right gluteus medius was greater in women than in men. We attribute this difference to several factors such as differences in muscle use during daily activities, asymmetries in physical exercise, or even genetic predisposition, handedness, leg dominance, and even subtle differences in hip structure. Muscle asymmetry between sides may be due to natural asymmetry in the body and movement patterns. In females, limb adduction may require higher gluteal medius and minimus muscle forces [80].

The findings of the present study provide an anatomy‐based chart that may aid in the management of painful disorders of the gluteal region. Approaches such as acupuncture, shock wave therapy, injections of local anesthetics, dry needling treatment, and other types of therapy have been used to alleviate symptoms caused by disorders related to MTPs, with clinical improvements being reported by some authors [81–88]. These techniques may benefit from additional anatomical knowledge; however, a better understanding of the physiopathology and diagnostic approaches to myofascial disorders is required.

### 4.1. Study Limitations

We were unable to form groups in terms of age and race, as the cadavers we had access to were not available to choose from, but were cadavers from the anatomy acquis. It is not possible to correlate MTPs in a living individual with the dissection using this method, as we cannot dissect in a living individual, which would lead to a clear understanding of the pathophysiology and diagnosis of myofascial disorders.

Because the number of donations is not large in our country, the statistician calculates a minimum sample size sufficient to reflect the general population according to the aim of the study, which in this case was to relate the SGN entry points to MTPs.

#### 4.1.1. Sample Size

The sample calculation was performed based on the results of the first six cadavers evaluated in a pilot study. The difference between Quadrants II and III (QII and QIII, respectively) was on average 2.17 nerve entry points in the pilot study, with a variability of 1.9 points (SD = 1.9 points). Thus, the sample size was calculated as 20 muscles, assuming a power of 78% and an alpha value of 5%.

#### 4.1.2. Cadaver Demographics

We were unable to form groups in terms of age and race, as the cadavers we had access to were not available to choose from, but were cadavers from the anatomy acquis. Because the number of donations is not large in our country, the statistician calculates a minimum sample size sufficient to reflect the general population according to the aim of the study, which in this case was to relate the SGN entry points to MTPs.

## 5. Conclusion

The pattern of distribution of the SGN branches has a topographical correspondence with MTPs.

## Conflicts of Interest

The authors declare no conflicts of interest.

## Author Contributions

All authors contributed to all stages of this study. Roberto Procópio Pinheiro: significant manuscript writer; study concept and design; anatomical dissection; and data acquisition. Daniela Andrea Medina Macaya: anatomy dissections; data acquisition; and technical procedures. Ana Maria Itezerote: anatomy dissections; data acquisition; and technical procedures. Samir Omar Saleh: anatomy dissections; study concept; and data interpretation. Flávio Hojaij: significant manuscript reviser; data acquisition; data analysis; and interpretation. Mauro Andrade: significant manuscript reviser; study concept and design; and data acquisition. Alfredo Luiz Jacomo: significant manuscript writing; study concept and design; data analysis; and interpretation. Flavia Emi Akamatsu Jacomo: significant manuscript revision; study concept and design; data acquisition; data analysis; and interpretation.

## Funding

No funding was received for this manuscript.

## Data Availability

The data that support the findings of this study are available on request from the corresponding author. The data are not publicly available due to privacy or ethical restrictions.
